# The natural history of protrusio acetabuli in Marfan syndrome and other hereditary connective tissue disorders: a 10-year follow-up CT study

**DOI:** 10.1186/s13023-025-03628-0

**Published:** 2025-03-12

**Authors:** Tordis Böker, Eva Kirkhus, Are Hugo Pripp, Svend Rand-Hendriksen, Benedicte Paus, Hans-Jørgen Smith, Rigmor Lundby

**Affiliations:** 1 Institute of Clinical Medicine, Faculty of Medicine, University of Oslo, Oslo, Norway; 2 Division of Radiology and Nuclear Medicine, Oslo University Hospital, Ullevål, PO Box 4956, Nydalen, Oslo, 0424 Norway; 3 Oslo Centre for Biostatistics and Epidemiology, Oslo University Hospital, Oslo, Norway; 4 TRS, National Resource Centre for Rare Disorders, Sunnaas Rehabilitation Hospital, Nesoddtangen, Norway; 5 Department of Medical Genetics, Oslo University Hospital, Oslo, Norway

**Keywords:** Computed tomography, Protrusio acetabuli, Marfan syndrome, Loeys-Dietz syndrome, Heritable ascending aortic diseases

## Abstract

**Objectives:**

To explore the natural history of protrusio acetabuli (PA) in adults with Marfan syndrome (MFS) via a prospective 10-year follow-up study.

**Methods:**

2014 through 2015, 62 of 87 survivors from a nationwide cross-sectional study of 105 adults with presumed MFS were re-examined. At follow-up, MFS was diagnosed in 46 participants, and other hereditary connective tissue disorders in 12 participants. As in the baseline study, matched hospital controls were collected for comparison. CT images were obtained of the hips. PA was evaluated quantitatively and qualitatively. Measurements were performed according to the circle-wall distance method. The data was analysed with paired t test, and McNemar’s test. A receiver operating characteristic (ROC) curve was constructed for the circle-wall distance.

**Results:**

There was no increase in the number of hips diagnosed with PA or in the circle-wall distance. PA was diagnosed in 58 of 87 hips in patients with MFS and in 71 of 111 hips in all patients with hereditary connective tissue disorders. Significantly more patients with MFS than controls had PA.

**Conclusion:**

The prevalence and degree of PA remained unchanged after 10 years. The circle-wall distance seems to have a good ability to discriminate between individuals with MFS and individuals without any known connective tissue disorder. Suggested cutoff level for the circle-wall distance: 2 mm.

**Clinical relevance:**

PA might be suggestive of a hereditary connective tissue disorder but does not develop or increase in adulthood. CT seems to have high sensitivity for PA and might be useful in a diagnostic process.

## Introduction

Marfan syndrome (MFS) is an autosomal dominant hereditary connective tissue disorder (HCTD) caused by mutation in *FBN1* [1]. As this gene exhibits significant allelic heterogeneity (MIM: 134797) [2], the diagnosis of MFS depends on diagnostic criteria that in addition to family history and molecular findings comprise findings in the cardiovascular system, eyes, skeleton, dura mater, lungs, and integument. Correct diagnosis and surveillance are important to prevent aortic aneurysm and other complications. As approximately 25% of MFS patients have no family history that would imply awareness of the disease [3], the increased and widespread use of diagnostic imaging may unintentionally raise the suspicion of the disorder. Knowledge about the prevalence of radiological features in MFS and in the general population is therefore important.

Protrusio acetabuli (PA) is a convex bulging of the acetabulum into the pelvis [4] and represents one of the skeletal features in the diagnostic criteria for MFS [5, 6]. Although it may be idiopathic, PA mostly occurs secondary to known disorders [7]. In addition to MFS, PA has been reported in other HCTDs, such as Loeys-Dietz syndrome (LDS), congenital contractural arachnodactyly and osteogenesis imperfecta [8, 9, 10]. The reported prevalence of PA in adults diagnosed with MFS varies from 16 to 100%, according to the chosen method for assessment of the pelvic girdle [11, 12, 13, 14, 15]. The prevalence of PA in other HCTDs is not known. Few studies have followed groups of MFS patients over a longer time span. Consequently, there is an evidence gap regarding when and how PA develops in patients with MFS or other HCTDs.

No gold standard for diagnosing PA has been established. Hence, different evaluation methods are in use. Conventional radiographs are frequently used to diagnose PA, although assessments of acetabula on conventional radiographs are prone to variations produced by differences in projections [16]. CT is not widely used to diagnose PA, although studies have shown that the circle-wall distance (CWD) measured on CT images is a reliable method [11, 13].

In this article, we present the results from a 10-year follow-up study on PA assessed with CT in a Norwegian cohort of patients with MFS and other HCTDs [13, 17]. We hypothesized that the number of patients diagnosed with PA and the degree of PA would increase over a time span of 10 years. The primary aim was to gain new information on how PA develops in adults with MFS by reinvestigating the morphology of the acetabula. The acetabula were evaluated in the same manner as at baseline; on CT images, measured by the CWD and visually [13].

A secondary aim was to re-evaluate the ability of PA to discriminate between patients with diagnosed MFS and controls by analysing the CWD cutoff value.

## Materials and methods

The follow-up study was approved by the Regional Committee for Medical and Health Research Ethics, Southeast Norway, registration number 2013/2109. All participants gave their informed consent.

### Study design

This is a 10-year follow-up of a nationwide cross-sectional study of adults with diagnosed or presumed MFS at baseline [13, 17]. Only survivors from the latter study were included. New matched controls were collected at follow-up, implying that the control groups at baseline and follow-up were independent.

### Study population

In 2003–2004 (baseline), 105 Norwegian adults (≥ 18 years) with presumed MFS attended a CT based case-control study on the prevalence of PA [13]. In December 2015, 18 of the original 105 patients had died.The 87 living patients were invited to attend in the follow-up study, and 62 gave their informed consent to participate. The cohort consisted of 42 women between 31 and 80 years of age (mean 49.2 ± 12.5 years, median 49.0 years) and 20 men between 30 and 65 years of age (mean 44.0 ± 8.9 years, median 43.5 years). Due to new diagnostic criteria and the availability of improved molecular techniques, the diagnosis was reassessed for all patients [6, 18]. In participants where no presumed disease-giving mutation had been found at baseline, high-throughput sequencing with a panel of 53 genes for connective tissue disorders was carried out. Reassessment with updated diagnostic criteria and high-throughput sequencing resulted in a change in diagnosis in 12 patients.

**Fig. 1:**
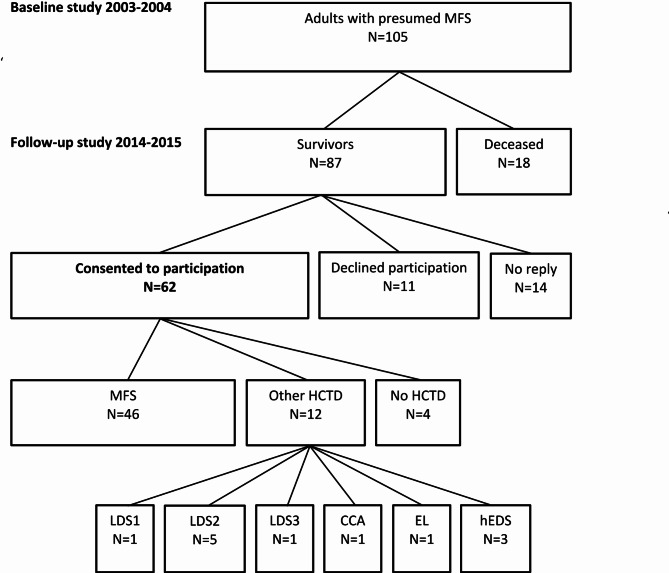
Cohort flowchart MFS, Marfan syndrome; LDS1, Loeys-Dietz syndrome type 1; LDS2, Loeys-Dietz syndrome type 2; LDS3, Loeys-Dietz syndrome type 3; CCA, congenital contractural arachnodactyly; EL, familial ectopia lentis; hEDS, Ehlers-Danlos syndrome hypermobile type

At follow-up, 46 patients were diagnosed with MFS (Table 1), seven with LDS (one LDS1, five LDS2, one LDS3), one with congenital contractural arachnodactyly (CCA), one with ectopia lentis (EL), and three with hypermobile Ehlers-Danlos syndrome (hEDS). Four out of 62 participants were excluded from further analyses due to no clinical or molecular indication of HCTD, leaving 58 eligible participants in this study (Fig. 1).

### Control population

A new control group was collected for the 10-year follow-up study. Selection criteria identical to those used at baseline were applied [13]. The controls were chosen from the pool of patients in the picture archiving and communication system (PACS) at our institution. CT of the pelvic area had been performed due to symptoms from the abdominal region or suspected vessel disease. Individuals with pronounced osteopenia or kidney/liver transplants were excluded. The controls were sex- and age-matched to the cohort and had no reported symptoms from the hips or known HCTD in the radiological request form. The mean and median age of the control group were in accordance with those of the HCTD cohort. The control group consisted of 64 individuals: 43 women between 31 and 78 years of age (mean 49.6 ± 12.5 years, median 49.0 years) and 21 men between 30 and 63 years of age (mean 44.0 ± 8.5 years, median 43.0 years) (Table 1).

### Imaging of the study group

At baseline, the CT examinations were performed with a GE Prospeed SX CT system (General Electric Medical Systems). During the 10 years the CT scanner was replaced. At follow-up, the CT examinations were performed with a Somatom Sensation 16 scanner (Siemens). The same CT protocol was used at baseline and 10-years follow-up. To keep the radiation dose as low as possible, only axial slices with 3 mm thickness were obtained through the acetabulum.

### Imaging of the control group

CT of the controls was performed with different CT scanners at our institution. Three mm thick axial reformations were used for evaluation.

### Measurements and definitions

**Fig. 2:**
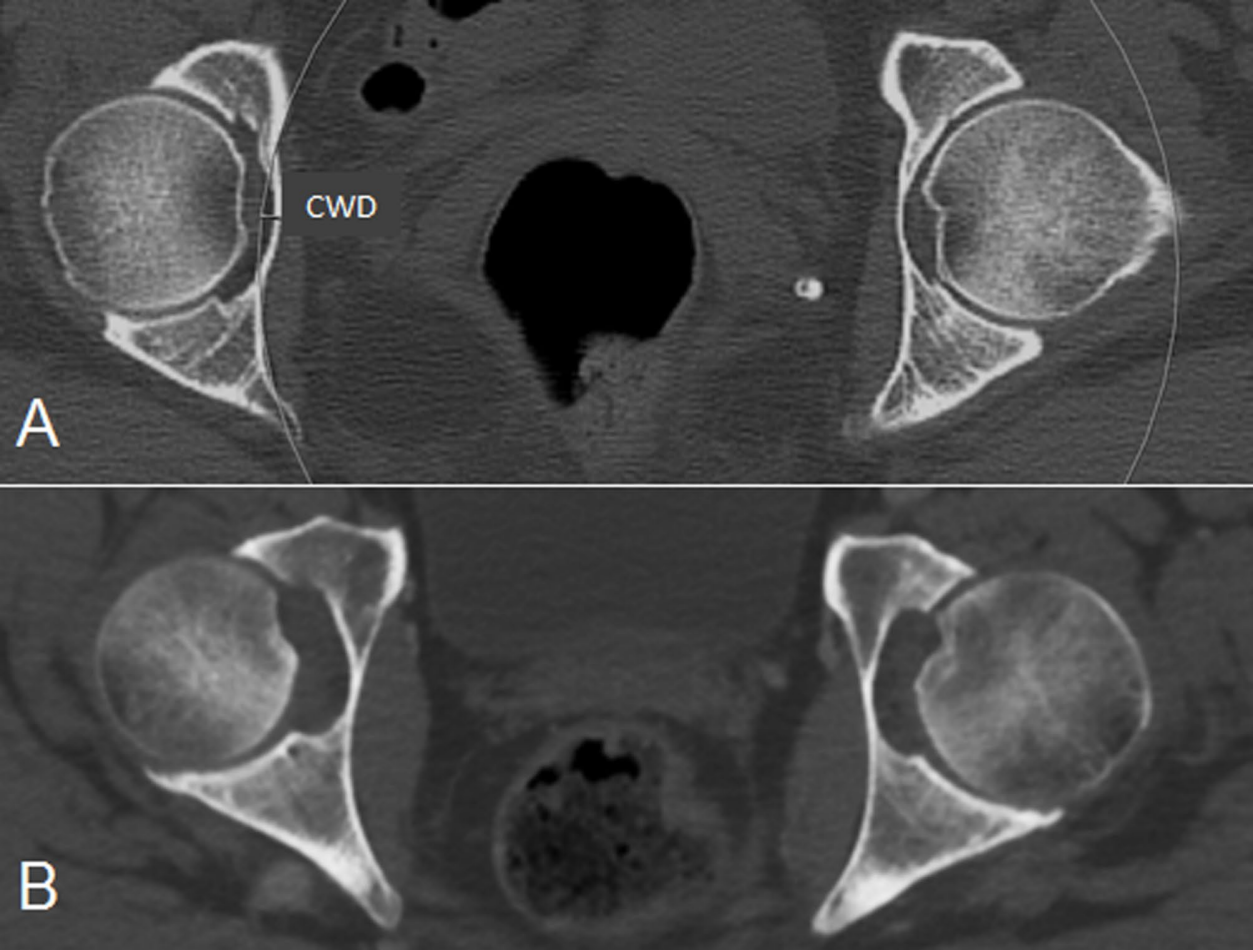
Axial CT images (3 mm thick sections) of the hips in two different patients. (**A**) Patient with Marfan syndrome and bilateral protrusio acetabuli. A circle with 10 cm radius was fitted to the inner pelvic wall of the right hip, and the degree of protrusion was measured as the distance between this circle and the most medial point of the inner pelvic wall of the acetabular fossa, the circle wall distance (CWD). (**B**) Normal hips for comparison

PA is defined as an inward bulging of the acetabulum into the pelvis [4]. An explorative CT based case-control study of PA in MFS patients was conducted at baseline [13]. In the present study, the same evaluation methods as in the first case-control study were used. For each subject, the assessment of PA was performed qualitatively and quantitatively on 3 mm axial bone reconstructions at the level of fusion between the acetabulum and ramus superior ossis pubis. Visually, the patients were diagnosed with PA if the acetabulum bulged into the pelvis on more than two subsequent images. The degree of protrusion was measured by the CWD method introduced by Lundby et al. at baseline (Fig. 2) [13]. The CWD values were dichotomized with cutoff values of 1, 2 and 3 mm to define PA in the hips.

In the cohort, prominent hip degeneration was registered; obliterated joint space, prominent osteophytes or subcortical cysts in the hips were noted. Hip replacements during the ten years were registered.

All assessments were performed in Sectra PACS, version 18.1.1 IDS7.

### Evaluation

Two musculoskeletal radiologists (TB and EK), both with more than 10 years of experience, evaluated all images in consensus. Both readers were unaware of the clinical status of the study patients but not blinded to which group (study or control) the patients belonged.

### Statistical analysis

Statistical analyses were performed for patients with MFS. Patients with other HCTDs were too few for statistical analyses, and the findings are reported with numbers (Table 2).

Five hips with total hip replacement were excluded from the statistical analyses and recorded as missing in the tables.

The data were checked for normality with histograms. The data were considered normally distributed in the cohort and skewed in the control groups. Continuous data were reported as mean and standard deviation or median and range (i.e., minimum – maximum) in accordance with the chosen analyses. Dichotomous data were reported as number of observations and percentage. Differences in the study group between baseline and follow-up were assessed with paired Student’s t test for continuous/discrete data and McNemar’s test for dichotomous data. Differences between the study group and the hospital controls were assessed with Mann-Whitney U test for continuous/discrete data and Chi-square test for dichotomized data. A receiver operating characteristic (ROC) curve was constructed for CWD.

For the statistical analyses, we used the Statistical Package for the Social Sciences, Version 27.0 (IBM SPSS Statistics).

## Results

**Table 1:** Characteristics of patients with MFS and controls at baseline and follow-up

Group	MFS		Controls	
Individuals	*N* = 46		Controls B *N* = 107	Controls F *N* = 64
Females N (%)	33 (72%)		68 (64%)	43 (67%)
	Baseline	Follow-up	Baseline	Follow-up
**Age**				
Mean ± SD Median	39.3 ± 11.8 38.0	50.1 ± 11.6 49.5	39.0 ± 13.3 37.0	47.8 ± 11.6 46.5
**Hips**	**n = 91 (1)**	**n = 87 (5)**	*n* = 214	*n* = 128

Whole numbers in parentheses: missing cases due to total hip replacement

No significant increase in CWD was found from baseline to follow-up in the patients with HCTDs or for the patients with MFS. For the patients with MFS, the CWD was at baseline, mean 2.80 (standard deviation 2.03), and at follow-up, mean 2.63 (standard deviation 1.94). There was no significant change in the number of MFS patients diagnosed with PA at follow-up compared to baseline irrespective of quantitative or qualitative assessment (Table 2).

**Fig. 3:**
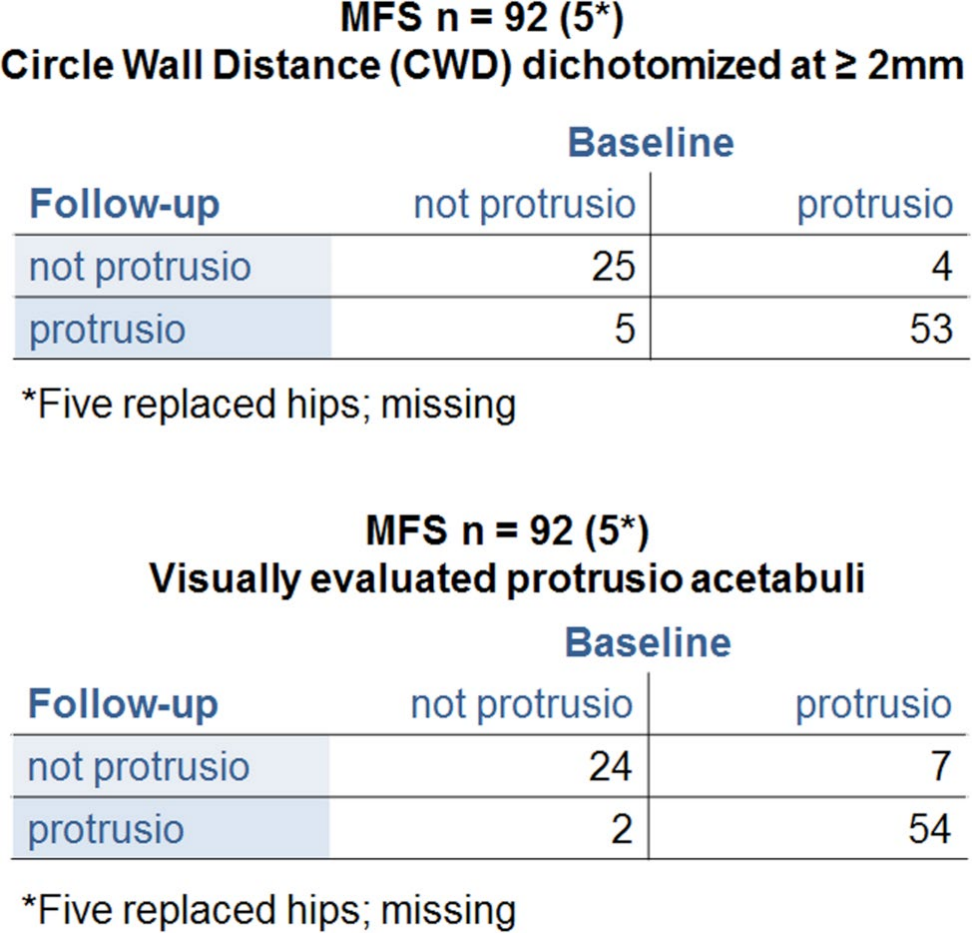
Cross tables: MFS patients at baseline and follow-up. Protrusio dichotomized at CWD ≥ 2 mm and visually

The number of hips in MFS patients diagnosed with PA at follow-up (58) was lower than that at baseline (61). This was also the case for hips in patients with other HCTDs (Table 2). Cross tables were made for the MFS patients (Fig. 3). When the assessment was made qualitatively, some patients lost the PA diagnosis at follow-up (Table 2).

**Table 2:** Prevalence of protrusio acetabuli in patients at baseline and follow-up

	Hereditary connective tissue disorder *N* = 58														
Individuals Hips	**MFS** *N* = 46 *n* = 92			**LDS1** *N* = 1 *n* = 2		**LDS2** *N* = 5 *n* = 10		**LDS3** *N* = 1 *n* = 2		**CCA** *N* = 1 *n* = 2		**EL** *N* = 1 *n* = 2		**hEDS** *N* = 3 *n* = 6	
	Baseline	Follow-up	Change *p*-value	B	F	B	F	B	F	B	F	B	F	B	F
**Hips with PA**															
CWD ≥ 3 mm^1)^	53; 58% (1)	50; 58% (5)	1.0	2	0	5	4	2	0	0	0	1	0	0	0
CWD ≥ 2 mm^1)^	61; 67% (1)	58; 67% (5)	1.0	2	2	6	6	2	2	0	2	2	1	0	0
CWD ≥ 1 mm^1)^	72; 79% (1)	68; 78% (5)	1.0	2	2	6	6	2	2	1	2	2	2	1	0
Qualitative assessment	65; 71% (1)	56; 64% (5)	0.18	2	0	6	6	2	0	0	2	2	2	0	0
Replaced hips	1; 1%	5; 5%	0.13	0	0	0	0	0	0	0	0	0	0	0	0
**Individuals with PA**															
CWD ≥ 3 mm^1)^	31; 67% (0)	28; 64% (2)	1.0	1	0	3	2	1	0	0	0	1	0	0	0
CWD ≥ 2 mm^1)^	35; 76% (0)	32; 73% (2)	1.0	1	1	3	3	1	1	0	1	1	1	0	0
CWD ≥ 1 mm^1)^	39; 85% (0)	37; 84% (2)	1.0	1	1	3	3	1	1	1	1	1	1	1	0
Qualitative assessment	35; 76% (0)	31; 71% (2)	0.63	1	0	3	2	1	1	0	1	1	1	0	0

Whole numbers in parentheses: missing cases due to total hip replacement

MFS, Marfan syndrome; LDS1, Loeys-Dietz syndrome type 1; LDS2, Loeys-Dietz syndrome type 2; LDS3, Loeys-Dietz syndrome type 3; hEDS, Ehlers-Danlos syndrome hypermobile type; CCA, congenital contractural arachnodactyly; EL, familial ectopia lentis; B, Baseline; F, Follow-up; CWD, circle wall distance; PA, protrusio acetabuli. ^1)^CWD dichotomized

Both at follow-up and baseline, the MFS patients had significantly larger CWD (*p* < 0.001) than the controls. (Table 3). Significantly more patients with MFS had PA than the controls, independent of the method or cutoff level used (*p* ≤ 0.001) (Table 3).

**Table 3:** Differences in CWD and PA between patients with MFS and controls at follow-up

Individuals Hips	MFS *N* = 46 *n* = 92	Controls *N* = 64 *n* = 128	Difference *p*-value
**CWD**			
Median Range mm	3.00 (5) (0–8)	0.00 (0–2)	< 0.001
**Hips With PA**			
CWD ≥ 3 mm^1)^	50; 58% (5)	0	< 0.001
CWD ≥ 2 mm^1)^	58; 67% (5)	8; 6%	< 0.001
CWD ≥ 1 mm^1)^	68; 78% (5)	28; 22%	< 0.001
Qualitative assessment	56; 64% (5)	14; 11%	< 0.001
Replaced hips	5; 5%	0	
**Individuals with PA**			
CWD ≥ 3 mm^1)^	28; 64% (2)	0	< 0.001
CWD ≥ 2 mm^1)^	32; 77% (2)	6; 9%	< 0.001
CWD ≥ 1 mm^1)^	37; 84% (2)	17; 27%	< 0.001
Qualitative	31; 71% (2)	10; 16%	< 0.001
Individuals with replaced hip	3; 7%	0	

Whole numbers in parentheses: missing cases due to total hip replacement

MFS, Marfan syndrome; CWD, circle wall distance; PA, protrusio acetabuli; ^1)^CWD dichotomized

**Fig. 4:**
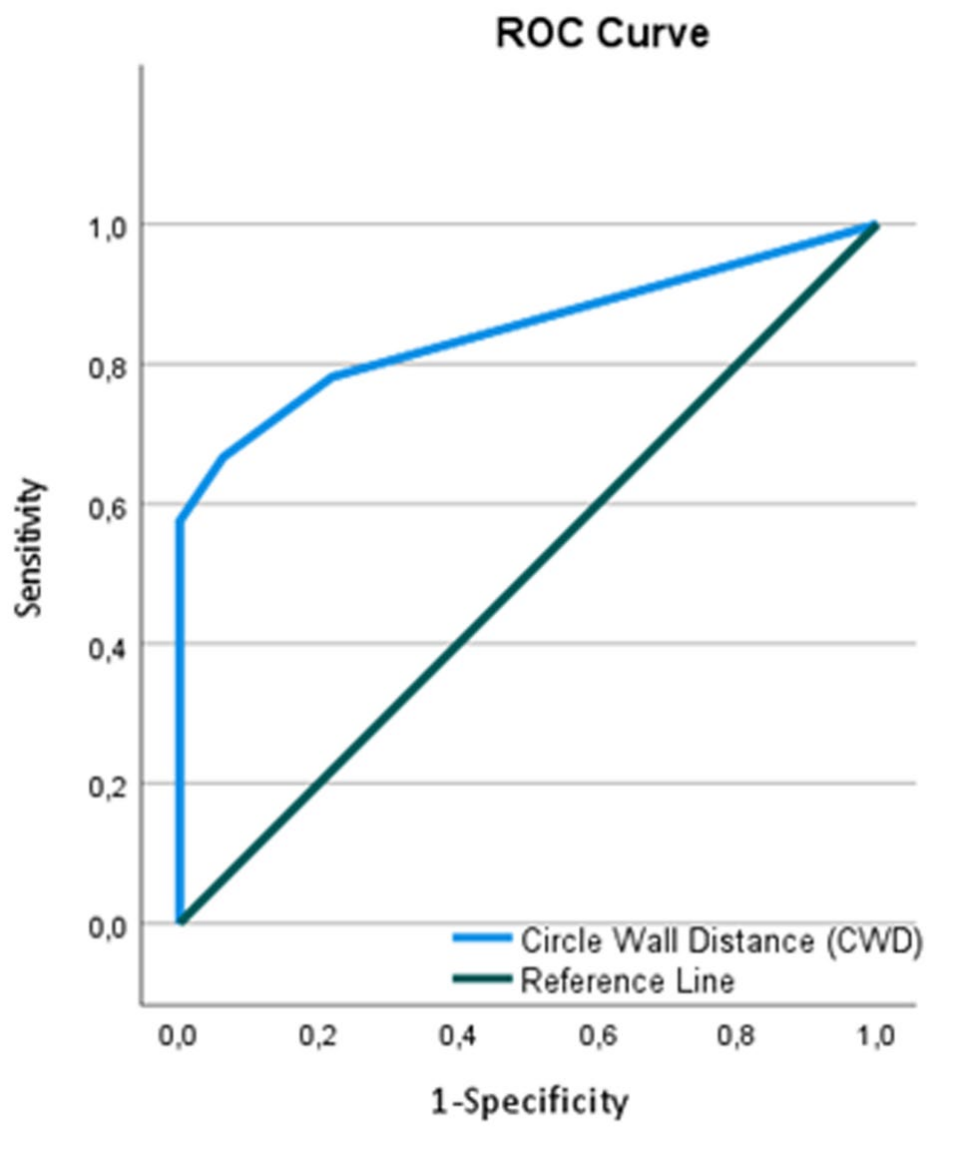
ROC analysis of CWD for MFS patients and controls at follow-up, AUC = 0.848

An ROC curve was constructed for CWD (Fig. 4). The area under the curve (AUC) was 0.848 (95% CI 0.8–0.91). As a marker for MFS CWD cutoff value of 1.5 mm resulted in sensitivity 66.7%, specificity 93.7%, positive predictive value (PPV) 87.9%, negative predictive value (NPV) 80.5%. CWD cutoff value of 2.5 mm resulted in sensitivity 57.5%, specificity 100%, PPV 100%, NPV 77.6%.

Three female MFS patients had total hip replacement in five hips at follow-up. In two of the patients, both hips were replaced during the follow-up period. The four hips had at the CT examinations from baseline osteophytes on the lateral acetabular rim and at the caput femoris margin and reduced joint space width, i.e., osteoarthritis Kellgren-Lawrence grade 3 or 4 [19, 20, 21]. One patient had total hip replacement in one hip at baseline, and the other hip was not replaced and had no pronounced degeneration.

We did not identify obliterated joint space, prominent osteophytes, or subchondral cysts in any of the participants at follow-up, i.e., no degenerative changes more than Kellgren-Lawrence grade 2 [19, 20, 21].

## Discussion

The results from our study support that PA is a frequent finding in adult MFS patients irrespective of age and remains stable. The lower absolute number of hips diagnosed with PA by CWD at follow-up in our study may be explained by measurement inaccuracy given by the placement of the endpoint cursors.

A high prevalence of PA (67%) both at follow-up and baseline in the patients diagnosed with MFS is in line with the findings of 78% PA in a Korean population of patients with MFS presented by Chun et al. in 2015, where the method introduced by Lundby and coworkers was used. The higher number of PA in Chun and coworkers’ study is probably explained by the lower cutoff value used, 1.25 mm.

The lack of a significant increase in the number of patients diagnosed with PA or in the degree of protrusion at follow-up in our study supports Sponseller and coworkers’ findings in their cross-sectional study from 2006, which concluded that the age-related prevalence of PA and hip function in MFS patients remained stable after the second decade of life. As noted by them, their study only provided limited information about the natural history of PA in MFS patients, as it was not longitudinal [14].

Our findings of PA in patients with other HCTDs except in patients with clinically diagnosed hEDS are in line with an earlier article on LDS1 and LDS2 from 2010, and no PA was reported in patients with hEDS [22, 23]. The findings support that PA may represent a sign of MFS and other HCTDs except for hEDS. However, our data on other HCTDs than MFS is small as the study is based on a cohort with presumed MFS at baseline.

The significant difference in CWD when MFS patients were compared to the controls led to further analyses of the CWD as a diagnostic tool by an ROC curve. AUC 0.848 (95% CI 0.8–0.91) indicates that CWD may be considered to have a good ability to discriminate between patients with MFS and patients without any known HCTD. PA might in combination with other skeletal signs of HCTD in some cases raise the suspicion of HCTD associated with thoracic aortic disease, such as MFS, before changes in the aorta can be detected [24, 25]. This underpins the choice of a cutoff value yielding a high sensitivity. The ROC analysis gave a sensitivity of 66.7% and specificity of 93.7% for a cutoff of 1.5 mm. Hence, a reasonable compromise between sensitivity and specificity seems to be given by a cutoff of 2 mm, which is also consistent with the qualitative assessment of PA (Table 2).

Conventional radiographs are still frequently used to diagnose PA, although it can be argued that CT has several advantages compared to conventional radiographs with respect to the diagnosis of PA. Earlier studies on PA in MFS where conventional radiographs were used are difficult to compare, as different criteria are used to define PA [14, 15, 26]. Furthermore, Richards and coworkers showed that the different methods used to diagnose PA on conventional radiographs are prone to variations depending on projection, tilt, and rotation [16]. The variation in measurements produced by tilt and rotation can be eliminated on CT and MRI reconstructions.

It is interesting that no early hip degeneration was found and that the prevalence of hip arthroplasty in patients with MFS in the cohort was lower than the estimated risk of total hip arthroplasty for osteoarthritis in the general population of Norway [27]. This observation is in line with the findings in a Danish register study on musculoskeletal diseases in MFS from 2022 [28]. Hence, regular follow-up imaging of the hips is not necessary in patients with MFS even though PA is a frequent finding.

### Limitations

This prospective cohort study has inherent limitations associated with loss of participants at follow-up. HCTDs are rare, and at follow-up, the cohort was relatively small. Some of the participants are related; therefore, the results should be interpreted with caution. Only the MFS group was large enough to allow conclusions with respect to changes during the follow-up. The fact that only 62 of the original 105 study participants were available for analysis, mainly due to death, possibly also to severe morbidity, represents a selection bias.

The hospital controls used at baseline were neither suitable nor available for re-examination after 10 years. Hence, a new control group had to be found at follow-up. Thus, the study design is not parallel, and changes in the study group are not parallel to the differences between the two control groups and hence not comparable. As different control groups were used at baseline and follow-up it is not possible to perform a paired test to analyse differences between the study group and the controls, and it is not possible to use a paired test to look for age related changes in the controls. The use of different control croups thereby might mask changes due to aging in controls.

Readings were performed by two radiologists in consensus; thus, interobserver reliability could not be tested. However, the CWD method has been tested for this in previous studies and shown a high interobserver agreement [11, 13]. Two observers performing the readings in consensus should result in uniformity. The CT scans of the study patients were done with axial slices through the acetabula to keep the radiation dose as low as possible. Therefore, it was difficult to blind the readers to which group (study or control) the subject belonged. This can be a source of bias, especially regarding the visual assessments, but the qualitative and quantitative evaluations correspond well, pointing against such a bias.

The only method available in this study for comparison with CWD was qualitative assessment of the hips on CT images. Radiographs were not obtained for comparison to keep the radiation dose as low as reasonable. MRI is not required to diagnose PA, as the diagnosis depends on the bony morphology of the acetabulum, but CT exposes the patients to radiation. With newer CT techniques such as iterative reconstruction, the radiation dose and scanning time are reduced. The radiation dose from CT scans with the use of ultralow dose techniques is comparable to that used with conventional radiographs [29, 30, 31].

### Future research perspectives

Symptoms from the hips were not correlated with our findings, and this remains to be explored. More studies on PA in children with suspected MFS are warranted to determine at what age PA develops. Prior publications on PA in other HCTDs than MFS including hEDS are very limited, and future research on the subject will probably benefit from international collaboration. Radiological research may in the future be designed to test AI-algorithms for automated detection of PA and other MFS-related manifestations on CT-investigations.

### Clinical considerations

PA may be a sign of MFS or another HCTD associated with thoracic aortic disease that requires surveillance.

One out of four patients diagnosed with MFS has no family history [3]. Therefore, it is important that radiologists and clinicians are aware that the finding of PA should raise the possibility of MFS, especially in the presence of other features of MFS, even in the absence of a positive family history. Patients with suspected MFS often have CT angiography of the aorta; this can be utilized to detect PA as part of a diagnostic process. Thus, in patients with suspected MFS, it seems reasonable to use CT to detect PA. On the other hand, PA is not known to predict early bony hip degeneration in patients with MFS, which leaves regular imaging of the hips after diagnosis unnecessary.

## Conclusion

The prevalence and degree of PA in MFS patients was high and remained unchanged after 10 years. In this study, CWD showed a good ability to discriminate between individuals with MFS and individuals without known HCTD. Suggested cutoff level for CWD: 2 mm.

## Data Availability

The raw data analysed during this study cannot be openly shared due to the need to protect individuals’ privacy under the General Data Protection Regulation. Data are available upon reasonable request to the corresponding author. The data are located in controlled access data storage at Oslo University Hospital.

## Abbreviations

CWD: Circle-wall distance
HCTD: Hereditary connective tissue disorder
hEDS: hypermobile Ehlers-Danlos syndrome
LDS: Loeys-Dietz syndrome
MFS: Marfan syndrome
NPV: Negative predictive value
PA: Protrusio acetabuli
PPV: Positive predictive value
ROC curve: Receiver operating characteristic curve
